# Effectiveness of the AVANT neurorehabilitation training program on stroke rehabilitation awareness and practice in Vietnam

**DOI:** 10.25122/jml-2026-0084

**Published:** 2026-08

**Authors:** Tuyen Van Nguyen, Linh Quang Nguyen, Ngoc Hoang Nguyen, Hoang Anh Le, Andreas Winkler

**Affiliations:** 1 Stroke Department, 108 Military Central Hospital, Hanoi, Vietnam; 2 EVER Neuro Pharma Asia Ltd., Hanoi, Vietnam; 3 MED SPACE, Korneuburg, Austria

**Keywords:** AVANT, post-stroke rehabilitation, stroke, neurorehabilitation, health education, caregivers, Vietnam, program evaluation, AVANT, Austrian Vietnamese Advancement Neurorehabilitation Treatment, BP, blood pressure, CI, confidence interval, CV, cardiovascular, EAN, European Academy of Neurology, EFNR, European Federation of NeuroRehabilitation Societies, MD, medical doctor, OT, occupational therapy, PT, physical therapy, Rx, prescription, WSO, World Stroke Organization

## Abstract

Stroke remains the leading cause of disability in Vietnam, yet awareness of stroke rehabilitation remains limited. The AVANT (Austrian Vietnamese Advancement Neurorehabilitation Treatment) Program, launched in 2017, aims to address this gap by training healthcare professionals and caregivers in motor recovery practices. This study evaluated the AVANT program's structure, content, and impact on stroke rehabilitation awareness, using a single-arm pre-post design without a control group for caregivers and a cross-sectional survey for healthcare professionals. Data were collected from 96 stroke units, each represented by one healthcare professional (Class A) and 3,352 caregivers (Class B). Post-training results showed significant improvements in knowledge and practices, with 90% of caregivers understanding the timing of rehabilitation initiation compared with 13.9% previously. The proportion of departments with no specific rehabilitation method fell from 58% to 0%, while the proportion following AVANT-specified exercises rose to 95% after training. These findings suggest that AVANT is associated with improved stroke rehabilitation knowledge and practices in Vietnam. As this was an observational study without a control group or direct patient outcome measures, further controlled studies are needed to confirm its impact on patient recovery.

## INTRODUCTION

Stroke is a leading cause of death and disability worldwide, with over 12.2 million new cases and more than 6.5 million deaths annually, imposing substantial economic and social burdens on patients, families, and health systems [1-4]. Vietnam, a developing country in Southeast Asia, has struggled to achieve expected healthcare system advancements. Over the past two decades, the incidence and mortality rates of stroke in Vietnam have risen steadily, disproportionately affecting younger populations and positioning stroke as the leading cause of death [5]. One key factor contributing to this alarming trend is the lack of public awareness and knowledge about stroke and post-stroke rehabilitation [6,7]. Awareness gaps delay diagnosis, emergency response, and treatment, significantly reducing patients’ chances of recovery. Furthermore, inadequate understanding of post-stroke rehabilitation exacerbates neural damage, prolongs recovery timelines, and increases treatment costs. Addressing these issues requires a strategic approach that emphasizes education on stroke prevention, management, and rehabilitation [8,9].

The establishment of the first stroke unit in Vietnam at the 108 Military Central Hospital in 2002 marked a critical milestone in stroke care. Over the subsequent 20 years, the country has expanded to more than 100 stroke units [5]. However, small-scale surveys indicate that public awareness of stroke remains limited [10]. Recognizing this gap, the AVANT Program was launched in 2017 as an international collaboration involving the World Stroke Organization, the Vietnam General Medical Association, the Vietnam Stroke Association, and the Vietnam Rehabilitation Association. The program aims to improve stroke patients’ reintegration into the community by enhancing the knowledge and understanding of healthcare professionals (Class A) and caregivers (Class B). AVANT provides comprehensive training in general stroke knowledge, prevention strategies, and basic rehabilitation techniques [11,12].

This study aimed to evaluate the current state of stroke treatment facilities in Vietnam, assess community awareness of stroke and rehabilitation, and provide a preliminary analysis of the AVANT program’s impact since its implementation. By identifying critical gaps, the AVANT program aims to lay the foundation for further advances in stroke care and rehabilitation.

## MATERIAL AND METHODS

### Study design and participants

This study combined a prospective, single-arm pre-post educational intervention design among caregivers with a cross-sectional survey of healthcare professionals, conducted between May 2020 and March 2023, to evaluate the impact of the AVANT program on stroke awareness and rehabilitation practices in Vietnam. The study involved two distinct participant groups: 96 stroke units across Vietnam, each represented by one healthcare professional respondent (AVANT-A class), and 3,352 caregivers of stroke patients (AVANT-B class). Healthcare professionals were selected from certified stroke units across Vietnam and had completed the AVANT program training between May 2020 and May 2022. Caregivers, aged 18 to 70 years, were recruited from five major stroke centers in Northern Vietnam, namely the 108 Military Central Hospital, Bach Mai Hospital, 103 Military Hospital, Phu Tho Provincial General Hospital, and Thanh Hoa Provincial General Hospital. Data collection for caregivers took place between May 2022 and March 2023 (Figure 1).

**Figure 1:**
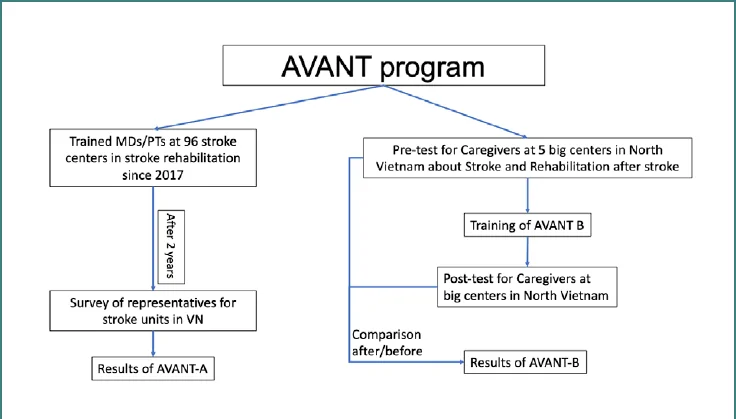
Diagram of the AVANT study

### Eligibility

The inclusion criteria required caregivers to be actively involved in emergency care, inpatient care, or post-discharge rehabilitation for stroke patients. Healthcare professionals were required to work in certified stroke units and to voluntarily consent to participate. Exclusion criteria included caregivers unable to read or understand the questionnaire and those who declined to participate in the study. Caregivers were primarily recruited through referrals from treating physicians at the participating hospitals as part of routine efforts to improve patient care; a smaller number learned about the AVANT program through educational videos or the program website and self-enrolled in the nearest available class. Systematic records of participation or refusal rates were not maintained and are therefore not reported; this is noted as a limitation below.

### The AVANT training program

The AVANT training program consisted of a 5-day course (8 hours per day, approximately 40 hours) that combined theoretical instruction, hands-on practical sessions led by rehabilitation technicians, and simulation-based practice with patients from the host hospital. All classes and locations used a standardized curriculum, including a textbook, lecture slides, handouts, and a practical training manual, appraised by the Vietnam Ministry of Health and published by the Medical Publishing House.

### Ethical considerations

This study was conducted as part of the routine evaluation of the AVANT training program, an ongoing educational and quality improvement initiative delivered within existing training activities. As the study involved a fully anonymized, voluntary knowledge and practice questionnaire, with no clinical intervention, no alteration of patient care, and no collection of identifiable patient information, formal review by an institutional ethics committee was not required, consistent with the exemption commonly applied to program evaluation and quality improvement activities in this setting. This determination was made by the study investigators; no institutional ethics committee or other regulatory body was formally consulted for a waiver or exemption. All participants, healthcare professionals and caregivers, were informed of the purpose of the survey and its voluntary, anonymous nature before completing the questionnaire, and participation was considered to imply consent. The study was conducted in accordance with the Declaration of Helsinki.

### Questionnaires

The Class A (healthcare professional) and Class B (caregiver) questionnaires were newly developed by the study team for the AVANT program, as no previously validated instrument covering this specific content was identified. Content was informed by consultation with senior rehabilitation and neurology specialists during development, and the questionnaires were pilot-tested at a stroke unit within the 108 Military Central Hospital prior to full-scale administration. One representative doctor from each participating department completed the Class A questionnaire, which combined individual clinical knowledge items with department-level practice indicators; caregivers completed the Class B questionnaire, a self-administered true/false and multiple-choice instrument, anonymously immediately before and after each training session. Formal reliability (e.g., Cronbach’s alpha) and validity testing were not conducted; this is acknowledged as a limitation below.

### Data collection and statistical analysis

Surveys were conducted before and after the AVANT training to assess participants’ knowledge and practices. Healthcare professionals completed surveys to evaluate their awareness of rehabilitation knowledge and its application within their facilities post-training. Caregivers answered true/false questions designed to assess their understanding of stroke risk factors, symptoms, timing of rehabilitation, and key practices such as patient positioning and aspiration prevention. We used a consecutive sampling technique for data collection. The collected data were processed using GraphPad Prism v10.0 (GraphPad Software, San Diego, CA, USA). We used descriptive statistics to present frequencies and percentages for qualitative variables and means for continuous variables. Inferential statistics, specifically chi-square tests, were used to compare pre- and post-training results, with statistical significance set at *P* < 0.05.

## RESULTS

### Current understanding of stroke in the community

Risk factors for stroke were correctly identified with frequencies ranging from 30.22% to 57.76%, with hypertension and cardiovascular disease being the most recognized (Figure 2A, Table 1). However, many risk factors were not considered, as indicated by a high rate of non-selections (Table 2).

**Figure 2:**
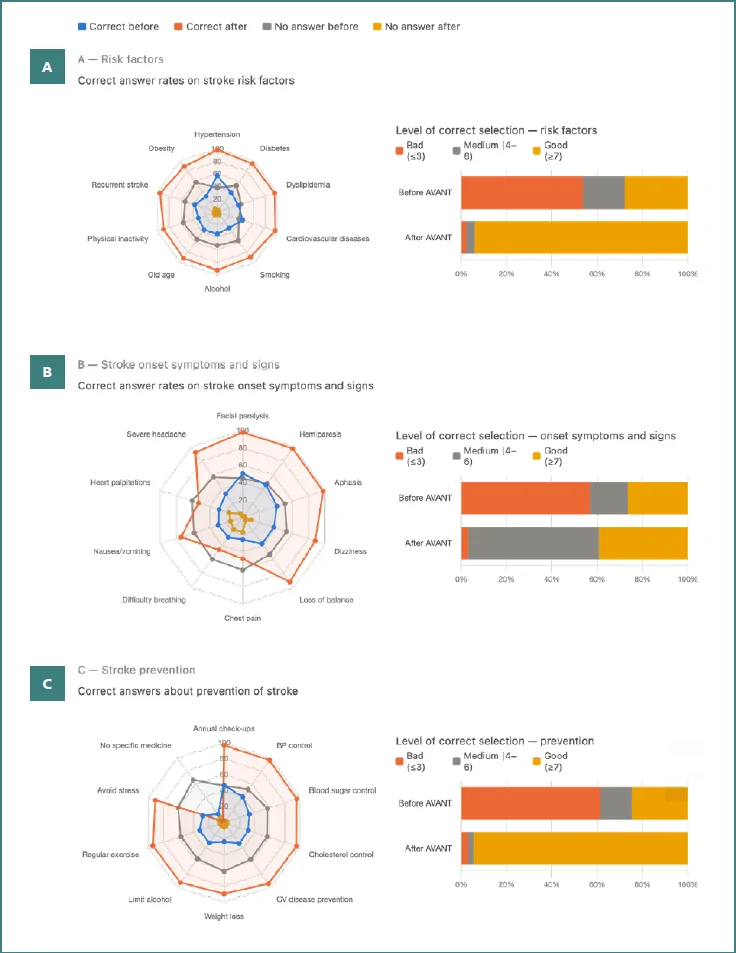
Changing perceptions of stroke risk factors, onset symptoms and signs, and prevention. Radar charts show the correct-answer rate for each individual item before and after the AVANT program; bar charts show the overall level of correct selections (bad, ≤ 3; medium, 4–6; good, ≥ 7). (A) Risk factors; (B) onset symptoms and signs; (C) prevention. Caregivers and healthcare professionals, *n* = 3352. Comparisons were made using the chi-square test; all *P* < 0.001.

**Table 1:** Caregiver recognition of stroke risk factors before and after the AVANT program

Measure	Before AVANT		After AVANT		*P*
	*n*	%	*n*	%	
**Risk factor recognized**					
Hypertension	1936	57.76%	3300	98.45%	*P* < 0.001
Diabetes	1260	37.59%	3174	94.69%	*P* < 0.001
Dyslipidemia	1190	35.50%	3212	95.82%	*P* < 0.001
Cardiovascular diseases	1417	42.27%	3245	96.81%	*P* < 0.001
Smoking	1064	31.74%	2993	89.29%	*P* < 0.001
Drinking (alcohol)	1183	35.29%	3108	92.72%	*P* < 0.001
Old age	1191	35.53%	3056	91.17%	*P* < 0.001
Physical inactivity	1062	31.68%	3006	89.68%	*P* < 0.001
Recurrent stroke	1264	37.71%	3203	95.55%	*P P* < 0.001
Obesity	1013	30.22%	2988	89.14%	*P* < 0.001
**Level of correct selection**					
Bad (≤3 correct)	1799	53.67%	71	2.12%	*P* < 0.001
Medium (4–6 correct)	619	18.47%	126	3.76%	*P* < 0.001
Good (≥7 correct)	934	27.86%	3155	94.12%	*P* < 0.001

AVANT, Austrian Vietnamese Advancement Neurorehabilitation Treatment. Caregivers, *n* = 3,352. Comparison of the proportion answering correctly before and after training using the chi-square test.

**Table 2:** Caregiver non-responses on stroke risk factors before and after the AVANT program

Measure	Before AVANT		After AVANT		*P*
	*n*	%	*n*	%	
Hypertension	1280	38.19%	47	1.40%	*P* < 0.001
Diabetes	1729	51.58%	109	3.25%	*P* < 0.001
Dyslipidemia	1332	39.75%	92	2.74%	*P* < 0.001
Cardiovascular diseases	1202	35.86%	81	2.42%	*P* < 0.001
Smoking	1895	56.53%	178	5.31%	*P* < 0.001
Drinking (alcohol)	1785	53.27%	116	3.46%	*P* < 0.001
Old age	1817	54.21%	190	5.67%	*P* < 0.001
Physical inactivity	1909	56.95%	185	5.52%	*P* < 0.001
Recurrent stroke	1805	53.85%	113	3.37%	*P* < 0.001
Obesity	1943	57.97%	200	5.97%	*P* < 0.001

AVANT, Austrian Vietnamese Advancement Neurorehabilitation Treatment. Caregivers, *n* = 3,352. Comparisons were made using the chi-square test.

For stroke onset symptoms, correct identification ranged from 33.35% to 50.36%. Misidentifications were common, with symptoms such as shortness of breath, chest pain, and palpitations being mistaken for stroke symptoms, with error rates between 10.35% and 13.19% (Figure 2B, Table 3). Many respondents left even typical symptoms, such as facial drooping and hemiplegia, unselected (Tables 3 and 4).

**Table 3:** Caregiver recognition of stroke onset signs before and after the AVANT program

Measure	Before AVANT		After AVANT		*P*
	*n*	%	*n*	%	
**Onset sign recognized**					
Facial paralysis	1688	50.36%	3282	97.91%	*P* < 0.001
Hemiparesis	1540	45.94%	3283	97.94%	*P* < 0.001
Aphasia	1393	41.56%	3262	97.32%	*P* < 0.001
Dizziness	1269	37.86%	2945	87.86%	*P* < 0.001
Loss of balance	1271	37.92%	3090	92.18%	*P* < 0.001
Chest pain†	442	13.19%	1171	34.93%	*P* < 0.001
Difficulty breathing†	400	11.93%	1210	36.10%	*P* < 0.001
Nausea/vomiting†	395	11.78%	2500	74.58%	*P* < 0.001
Heart palpitations†	347	10.35%	1005	29.99%	*P* < 0.001
Severe headache	1118	33.35%	3097	92.39%	*P* < 0.001
**Level of correct selection**					
Bad (≤3 correct)	1900	56.68%	103	3.07%	*P* < 0.001
Medium (4–6 correct)	562	16.77%	1930	57.58%	*P* < 0.001
Good (≥7 correct)	890	26.55%	1319	39.35%	*P* < 0.001

AVANT, Austrian Vietnamese Advancement Neurorehabilitation Treatment. Caregivers, *n* = 3,352. Comparisons were made using the chi-square test. † Non-stroke symptom, included to assess misidentification; a higher value therefore indicates poorer performance.

**Table 4:** Caregiver non-responses on stroke onset signs before and after the AVANT program

Measure	Before AVANT		After AVANT		*P*
	*n*	%	*n*	%	
Facial paralysis	1500	44.75%	54	1.61%	*P* < 0.001
Hemiparesis	1604	47.85%	60	1.79%	*P* < 0.001
Aphasia	1717	51.22%	71	2.12%	*P* < 0.001
Dizziness	1786	53.28%	334	9.96%	*P* < 0.001
Loss of balance	1773	52.89%	179	5.34%	*P* < 0.001
Chest pain	2045	61.01%	581	17.33%	*P* < 0.001
Difficulty breathing	1995	59.52%	587	17.51%	*P* < 0.001
Nausea/vomiting	1964	58.59%	496	14.80%	*P* < 0.001
Heart palpitations	2048	61.10%	553	16.50%	*P* < 0.001
Severe headache	1924	57.40%	160	4.77%	*P* < 0.001

AVANT, Austrian Vietnamese Advancement Neurorehabilitation Treatment. Caregivers, *n* = 3,352. Comparisons were made using the chi-square test.

When asked about first aid measures for suspected stroke cases, only 24.25% indicated they would call emergency services (115, the national emergency number in Vietnam). Notably, 3.96% opted for Gua Sha (skin scraping) and 19.12% chose fingertip bloodletting as initial responses. Over half of respondents (50.52%) did not select any first-aid measure (Figure 3, Table 5).

**Figure 3:**
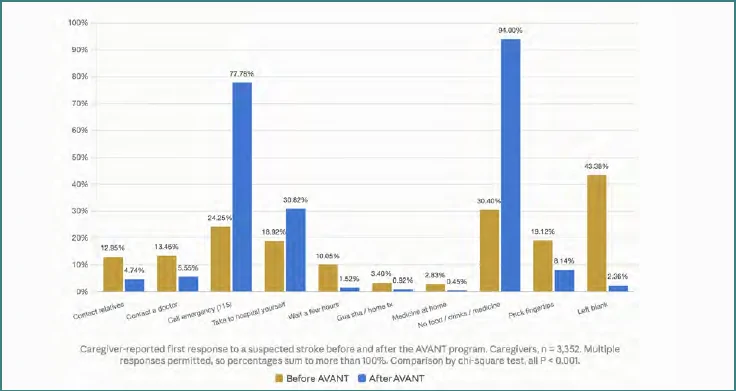
Caregiver-reported first response to a suspected stroke before and after the AVANT program. Caregivers, *n* = 3352. Comparisons were made using the chi-square test; all *P* < 0.001.

**Table 5:** Caregiver-reported first response to a suspected stroke before and after the AVANT program

Measure	Before AVANT		After AVANT		*P*
	*n*	%	*n*	%	
**Response chosen**					
Contact patient’s relatives	434	12.95%	159	4.74%	*P* < 0.001
Contact a doctor you know	451	13.46%	186	5.55%	*P* < 0.001
Call emergency services (115)	813	24.25%	2607	77.78%	*P* < 0.001
Take patient to hospital yourself	634	18.92%	1033	30.82%	*P* < 0.001
Wait a few hours then go	337	10.05%	51	1.52%	*P* < 0.001
Gua sha / home treatment	114	3.40%	31	0.92%	*P* < 0.001
Take medicine at home (no Rx)	95	2.83%	15	0.45%	*P* < 0.001
No food/drinks/medicine	1019	30.40%	3151	94.00%	*P* < 0.001
Prick fingertips to let blood	641	19.12%	273	8.14%	*P* < 0.001
Left blank / no selection	1454	43.38%	79	2.36%	*P* < 0.001

AVANT, Austrian Vietnamese Advancement Neurorehabilitation Treatment. Caregivers, *n* = 3,352. Multiple responses permitted, so percentages sum to more than 100%. Comparisons were made using the chi-square test.

For stroke prevention, correct identification rates ranged from 24.22% (weight loss) to 47.02% (annual health check-ups). Misconceptions persisted, with 12.92% believing that taking a specific medication could prevent stroke (Figure 2C, Tables 6 and 7).

**Table 6:** Caregiver knowledge of stroke prevention before and after the AVANT program

Measure	Before AVANT		After AVANT		*P*
	*n*	%	*n*	%	
**Prevention measure identified**					
Annual health check-ups	1576	47.02%	3255	97.11%	*P* < 0.001
Blood pressure control	1336	39.86%	3242	96.72%	*P* < 0.001
Blood sugar control	1126	33.60%	3213	95.85%	*P* < 0.001
Cholesterol control	1074	32.04%	3202	95.53%	*P* < 0.001
Treatment/prevention of CV	1090	32.52%	3179	94.84%	*P* < 0.001
Weight loss	812	24.22%	2994	89.32%	*P* < 0.001
Limit alcohol intake	1053	31.41%	3125	93.23%	*P* < 0.001
Regular exercises	1084	32.34%	3143	93.79%	*P* < 0.001
Avoid stress	945	28.19%	3037	90.60%	*P* < 0.001
Take one special medicine only	433	12.92%	89	2.66%	*P* < 0.001
**Level of correct selection**					
Bad (≤3 correct)	2054	61.28%	102	3.04%	*P* < 0.001
Medium (4–6 correct)	468	13.96%	83	2.48%	*P* < 0.001
Good (≥7 correct)	830	24.76%	3167	94.48%	*P* < 0.001

AVANT, Austrian Vietnamese Advancement Neurorehabilitation Treatment; CV, cardiovascular. Caregivers, *n* = 3,352. Comparison using chi-square test.

**Table 7:** Caregiver non-responses on stroke prevention before and after the AVANT program

Measure	Before AVANT		After AVANT		*P*
	*n*	%	*n*	%	
Annual health check-ups	1557	46.45%	87	2.60%	*P* < 0.001
Blood pressure control	1723	51.40%	95	2.83%	*P* < 0.001
Blood sugar control	1925	57.45%	121	3.61%	*P* < 0.001
Cholesterol control	1908	56.92%	130	3.88%	*P* < 0.001
Treatment/prevention of CV	1911	57.01%	151	4.50%	*P* < 0.001
Weight loss	2050	61.16%	209	6.24%	*P* < 0.001
Limit alcohol intake	1886	56.26%	165	4.92%	*P* < 0.001
Regular exercises	1905	56.83%	176	5.25%	*P* < 0.001
Avoid stress	2036	60.74%	235	7.01%	*P* < 0.001
Take one special medicine only	2217	66.14%	267	7.97%	*P* < 0.001

AVANT, Austrian Vietnamese Advancement Neurorehabilitation Treatment; CV, cardiovascular. Caregivers, *n* = 3,352. Comparisons were made using the chi-square test.

### Current state of Vietnamese post-stroke rehabilitation

Caregivers demonstrated limited understanding of rehabilitation before participating in the AVANT program. Only 37.08% believed rehabilitation was necessary post-stroke (Table 8), and 26.73% thought rehabilitation was the shared responsibility of medical staff and caregivers, while 14.41% believed it was solely the responsibility of medical staff (Figure 4A). Knowledge about the timing of rehabilitation and basic practices was also not well established: only 13.48% identified the correct time to start rehabilitation (24-48 hours post-onset, depending on the patient’s stability post-stroke), and 27.27% knew that repositioning patients every 2 hours is essential (Figure 4B and 4C).

**Table 8:** Caregiver perceptions of post-stroke rehabilitation before and after the AVANT program

Measure	Before AVANT		After AVANT		*P*
	*n*	%	*n*	%	
**Who should perform rehabilitation exercises**					
Medical staff only	483	14.41%	66	1.97%	*P* < 0.001
Caregivers only	347	10.35%	108	3.22%	*P* < 0.001
Medical staff AND caregivers	896	26.73%	3145	93.83%	*P* < 0.001
No answer	1716	51.19%	130	3.88%	*P* < 0.001
**Should stroke patients do rehabilitation exercises**					
NO	757	22.58%	78	2.33%	*P* < 0.001
YES	1243	37.08%	3162	94.33%	*P* < 0.001
No answer	1352	40.33%	112	3.34%	*P* < 0.001
**Impact of rehabilitation exercises**					
Recover motor functions (PT)	399	11.90%	111	3.31%	*P* < 0.001
Daily activities independently (OT)	315	9.40%	67	2.00%	*P* < 0.001
Improve speech/comprehension	301	8.98%	33	0.98%	*P* < 0.001
Safe and comfortable eating	168	5.01%	23	0.69%	*P* < 0.001
All of the above	663	19.78%	3099	92.45%	*P* < 0.001
No answer	1798	53.64%	108	3.22%	*P* < 0.001
**When to start motor rehabilitation**					
After 3–5 days	336	9.74%	90	2.62%	*P* < 0.001
Within 24–48 h (if doctor allows)	465	13.48%	3022	87.82%	*P* < 0.001
After 1 week (when stable)	436	12.64%	125	3.63%	*P* < 0.001
After 2 weeks / post-discharge	361	10.47%	60	1.74%	*P* < 0.001
No answer	1851	53.67%	144	4.18%	*P* < 0.001
**Does the unparalyzed side need practice**					
NO	771	24.46%	1101	32.85%	*P* < 0.001
YES	960	30.46%	2126	63.42%	*P* < 0.001
No answer	1421	45.08%	125	3.73%	*P* < 0.001
**A paralyzed patient should lie only on the back or the healthy side**					
TRUE (incorrect belief)	1448	43.19%	953	28.42%	*P* < 0.001
FALSE (correct)	406	12.11%	2261	67.43%	*P* < 0.001
No answer	1499	44.71%	139	4.15%	*P* < 0.001
**How often should the patient be repositioned**					
Once every 2 hours	914	27.27%	2662	79.42%	*P* < 0.001
Once every 5 hours	338	10.08%	112	3.34%	*P* < 0.001
Once every 8 hours	237	7.07%	25	0.75%	*P* < 0.001
No answer	1987	59.28%	566	16.89%	*P* < 0.001

AVANT, Austrian Vietnamese Advancement Neurorehabilitation Treatment; OT, occupational therapy; PT, physical therapy. Comparisons were made using the chi-square test.

**Figure 4:**
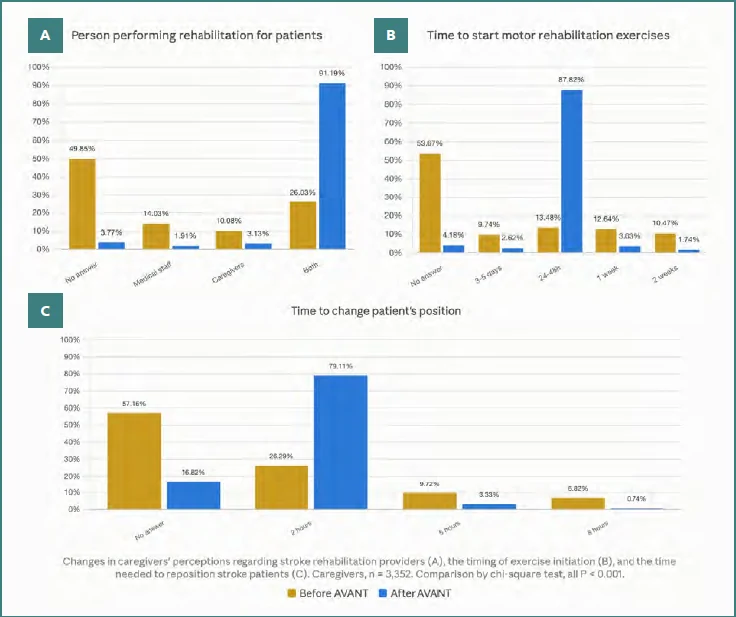
Changes in caregivers' perceptions of stroke rehabilitation. (A) Rehabilitation providers; (B) timing of exercise initiation; (C) time needed to reposition patients with stroke. Caregivers, *n* = 3352. Comparisons were made using the chi-square test; all *P* < 0.001.

### Responsibility for patients’ rehabilitation

Before AVANT, rehabilitation was not included in the stroke treatment protocol in 17% of stroke units. Early rehabilitation within 3 days of stroke onset occurred in just 30% of cases, while 24% started after one week and 29% initiated rehabilitation only after discharge (Figure 5AB). Rehabilitation was delivered in the stroke department in 39% of units and in a dedicated neurorehabilitation department in 48% (Figure 5C). Moreover, only 73% of medical staff recognized the benefit of combining neurotrophic agents, such as Cerebrolysin, with rehabilitation therapy [13], and just 35% reported combining Cerebrolysin specifically (Figure 5D).

**Figure 5:**
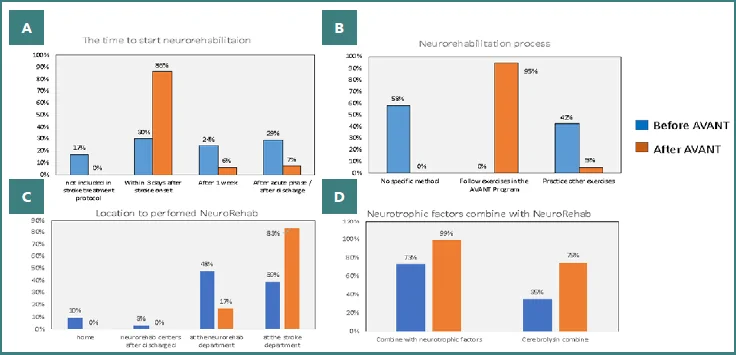
Stroke-unit practice before and after the AVANT program. (A) Timing of neurorehabilitation; (B) rehabilitation method used; (C) location of delivery; (D) combination with neurotrophic agents. Stroke units, *n* = 96.

### Effectiveness of the AVANT program

The AVANT program significantly improved stroke caregivers’ and medical professionals’ knowledge. Correct identification of stroke risk factors increased from 30.22%–57.76% before training to 89.14%–98.45% after training, with poor knowledge rates dropping from 53.67% to 2.12%. Awareness of stroke symptoms and appropriate emergency responses, such as calling emergency services, rose from 24.25% to 77.78%, while unscientific practices such as Gua Sha decreased significantly (Figure 3, Table 5). Among medical staff, the proportion correctly identifying the timing to start rehabilitation increased from 30% to 86% (Figure 5A).

Knowledge of aspiration-prevention techniques improved similarly. Correct identification of safe eating position (sitting rather than lying), head posture (chin lowered), and head orientation (turned to the paralyzed side) rose from 40.29%, 33.27%, and 19.03% before training to 64.20%, 58.54%, and 53.00% after training, respectively. At the same time, recognition of soft, pudding-textured food as appropriate for patients with swallowing difficulty increased from 9.97% to 62.79% (Table 9). Non-response rates on these items also fell sharply, from over 40% before training to under 5% after (Table 9).

**Table 9:** Caregiver knowledge of rehabilitation technique and aspiration prevention before and after the AVANT program

Measure	Before AVANT		After AVANT		*P*
	*n*	%	*n*	%	
**Rehabilitation technique**					
Do not put weight on paralyzed arm/leg	750	22.37%	2456	73.27%	*P* < 0.001
Paralyzed side: passive exercises only	1021	30.46%	2225	66.38%	*P* < 0.001
Put sleeve on paralyzed side first	726	21.66%	2944	87.83%	*P* < 0.001
Walk up stairs: paralyzed leg first	1086	32.40%	2591	77.30%	*P* < 0.001
Walk down stairs: paralyzed leg first	424	12.65%	1767	52.71%	*P* < 0.001
**Body position while eating**					
Lying	581	17.33%	1092	32.58%	*P* < 0.01
Sitting	1351	40.29%	2152	64.20%	*P* < 0.01
No answer	1421	42.38%	108	3.22%	*P* < 0.001
**Head position while eating**					
Head looking up (chin up)	763	22.75%	1263	37.67%	*P* < 0.001
Chin lowered / head looking down	1116	33.27%	1963	58.54%	*P* < 0.001
No answer	1475	43.98%	127	3.79%	*P* < 0.001
**Head rotation while eating**					
Head turned to paralyzed side	638	19.03%	1777	53.00%	*P* < 0.001
Head turned to healthy side	1078	32.15%	1398	41.69%	*P* < 0.001
No answer	1637	48.82%	178	5.31%	*P* < 0.001
**Food texture for swallowing disorder**					
Liquid food	920	27.45%	1098	32.76%	*P* < 0.001
Soft food (pudding texture)	336	10.02%	2119	63.21%	*P* < 0.001
Solid food	82	2.45%	22	0.66%	*P* < 0.001
No answer	2032	60.62%	136	4.06%	*P* < 0.001

AVANT, Austrian Vietnamese Advancement Neurorehabilitation Treatment. Caregivers, *n* = 3,352. Comparisons were made using the chi-square test.

## DISCUSSION

The present study demonstrates that the AVANT program has been an effective educational intervention to strengthen stroke rehabilitation awareness and practices in Vietnam. Before the program’s implementation, both caregivers and healthcare professionals exhibited significant knowledge gaps regarding stroke prevention, recognition, and rehabilitation. The AVANT program addressed many of these shortcomings, producing measurable gains in understanding, behavior, and clinical application. These findings highlight that structured educational initiatives can play a pivotal role in improving post-stroke recovery outcomes in low- and middle-income settings.

### Baseline gaps in awareness and practice

Before AVANT, misconceptions about stroke were widespread in the Vietnamese community. Traditional first-aid methods such as Gua Sha (skin scraping) and fingertip bloodletting remained common, and few respondents identified calling emergency services as the appropriate first response (Table 3). These misconceptions mirror findings from other developing countries, where cultural habits and limited access to health education impede timely recognition and emergency care [14].

Among healthcare professionals, variation in baseline knowledge reflected the uneven distribution of specialized stroke care across regions. While the number of certified stroke units has expanded rapidly in Vietnam, integration of early and structured rehabilitation remains inconsistent. Rehabilitation often begins late or is fragmented between departments, delaying neural recovery and increasing the risk of complications such as aspiration pneumonia, pressure ulcers, and muscle contractures [15].

### Impact of the AVANT program

The AVANT program demonstrated significant improvements in both knowledge and applied practice. Among caregivers, the rate of correct emergency response increased more than threefold, and reliance on unscientific traditional treatments dropped sharply. This change is particularly important because caregivers often serve as first responders in Vietnam, where pre-hospital emergency systems are still developing. Improved knowledge of correct positioning, aspiration prevention, and early mobilization directly influences patient recovery and quality of life.

Among healthcare professionals, the proportion of departments with no specific rehabilitation method fell from 58% to 0%, while 95% followed AVANT-specified exercises after training, indicating both successful knowledge transfer and behavioral change within clinical teams. The improved awareness of combining rehabilitation with neurotrophic support, such as Cerebrolysin, is consistent with the European Academy of Neurology and European Federation of NeuroRehabilitation Societies (EAN/EFNR) guideline, which recommends Cerebrolysin as pharmacological support for early motor rehabilitation after acute ischemic stroke and reflects established practice in several European countries [16]. As this study did not directly evaluate Cerebrolysin’s efficacy or patient-level neurorecovery outcomes, this finding should be interpreted as improved decision-making among clinicians rather than evidence of a treatment effect. Importantly, AVANT encouraged a multidisciplinary approach linking neurologists, physiotherapists, and rehabilitation specialists, which is essential for sustainable outcome improvements.

### Comparison with other educational models

Educational programs targeting stroke awareness have shown encouraging but variable results elsewhere in the region. In South India, a school- and college-based community education program significantly improved recognition of stroke signs and reduced reliance on traditional remedies as an initial response. However, the intervention targeted general public awareness rather than caregivers of stroke patients specifically [14]. More broadly, stroke education and prehospital response programs in other low- and middle-income countries have shown similar knowledge gains, though sustained integration into clinical practice remains a common challenge, particularly where pre-hospital emergency systems are still developing [17]. Compared with these programs, AVANT combined dual-targeted training, addressing both professionals and caregivers, within a standardized, Ministry of Health–approved curriculum delivered consistently across multiple sites, which may explain its broad reach.

### Remaining challenges

Despite these achievements, several challenges persist. First, heterogeneity in baseline education levels among caregivers limited the depth of knowledge acquisition for some participants. Rural and remote areas continue to have fewer trained rehabilitation professionals, and follow-up supervision after training remains limited. Extending AVANT’s duration or introducing refresher courses could help maintain gains over time.

Second, many hospitals have not fully integrated rehabilitation protocols into stroke unit workflows. Structural barriers, such as shortages of physiotherapists, lack of dedicated space, and inconsistent use of standardized outcome measures, continue to hinder progress. Moreover, societal attitudes toward disability and reliance on family-based care may delay patients’ return to structured therapy programs [18].

Third, while AVANT primarily addressed motor recovery and included basic caregiver-level awareness of aspiration-prevention practices, other highly prevalent post-stroke complications, such as formal dysphagia screening and management, aphasia, cognitive deficits, and occupational therapy needs, were beyond its initial scope.

### Implications for future programs

The outcomes of this study suggest the feasibility of targeted, scalable educational interventions for stroke rehabilitation. The results support launching an expanded AVANT II project that builds on the current foundation while addressing additional domains of post-stroke dysfunction. AVANT II should incorporate specialized modules on swallowing rehabilitation, communication therapy, cognitive retraining, and activities of daily living.

Digital learning tools and tele-rehabilitation could also expand reach, especially in underserved rural areas. Integration with national stroke care networks and ongoing collaboration between Vietnamese and international rehabilitation experts would ensure sustainability.

### Strengths and limitations

A major strength of this study is its large, multicenter sample, which includes both healthcare professionals and lay caregivers, allowing a comprehensive assessment of stroke awareness. Additionally, the program’s dual training design allowed evaluation of knowledge transfer at both clinical and community levels.

However, several limitations warrant acknowledgment. The observational design lacked a control group, limiting causal inference. The study did not systematically record caregiver recruitment, participation, and refusal rates, precluding assessment of potential selection bias. Self-reported data may introduce response bias; in addition, the Class A and Class B questionnaires were newly developed for this program and pilot tested, but did not undergo formal reliability or validity testing. Department-level practice indicators reported by Class A representatives were based on self-estimation by a single representative per department rather than independent audit, which may be subject to recall or estimation bias. Future studies should incorporate validated instruments, formal psychometric evaluation, and objective practice audits where feasible, as well as longitudinal follow-up to assess whether knowledge improvements translate into sustained behavior change and better patient outcomes. In addition, denominators varied across individual survey items because respondents could leave any question unanswered, which should be considered when interpreting item-level percentages.

## CONCLUSION

This study suggests that the AVANT program is associated with meaningful improvements in stroke-related knowledge, attitudes, and self-reported practices among healthcare professionals and caregivers in Vietnam. Participants showed clearer understanding of early rehabilitation timing, basic positioning and aspiration-prevention practices, and a marked reduction in reliance on unscientific traditional practices. Given the observational, single-arm design of this study, these findings should be interpreted as an association between AVANT participation and improved knowledge and practice, rather than definitive proof of the program’s effectiveness in changing patient outcomes; no direct measures of patient functional recovery, disability, quality of life, or long-term reintegration were collected.

The current AVANT program focused on motor recovery and included basic caregiver-level awareness of aspiration-prevention practices. Other common post-stroke complications, including formal dysphagia screening and management, aphasia, and cognitive impairment, were outside its scope and remain important gaps in stroke rehabilitation education in Vietnam. Building on the knowledge gains described in this manuscript, an AVANT II initiative is planned to extend training into these areas and to incorporate digital and tele-rehabilitation formats to reach rural and underserved regions. Future work should include a control group and direct patient outcome measures to evaluate the program’s impact on stroke recovery more rigorously.

## Data Availability

The datasets generated and analyzed during the current study are available from the corresponding author on reasonable request.

## Personal thanks

The authors acknowledge the Vietnam Stroke Association, the World Stroke Organization, and participating hospitals for their support and collaboration in implementing the AVANT program.
